# “Why me? I don’t fit the mould … I am a freak of nature”: a qualitative study of women’s experience of gout

**DOI:** 10.1186/s12905-015-0277-z

**Published:** 2015-12-28

**Authors:** Jane C. Richardson, Jennifer Liddle, Christian D. Mallen, Edward Roddy, Suman Prinjha, Sue Ziebland, Samantha Hider

**Affiliations:** Research Institute for Primary Care and Health Sciences, Keele University, Keele, Staffordshire ST5 5BG UK; Nuffield Department of Primary Care Health Sciences, University of Oxford, Gibson Building, 1st Floor, Radcliffe Observatory Quarter, Woodstock Road, Oxford, OX2 6GG UK

**Keywords:** Gout, Inflammatory arthritis, Qualitative research, Patient experience

## Abstract

**Background:**

Gout is more common in men, and is often perceived by both patients and health practitioners to be a disorder of men, but its prevalence in women is increasing. Little is known about women’s experience of gout and the impact it has on their lives. It is important for practitioners to be aware of these areas, given the increasing numbers of women with gout they are likely to see in the future. This study aimed to explore women’s experiences of gout.

**Methods:**

A qualitative research design was used. Semi-structured interviews were conducted with 43 people, of whom 14 were women. Interviews were video and/or tape recorded and transcribed verbatim. Data from the interviews was first grouped into broad categories, followed by a more detailed thematic analysis and interpretation.

**Results:**

Participants’ ages ranged from 32 to 82. Nine participants were retired and five were in fulltime work. Four themes emerged: (1) experience of onset, help seeking and diagnosis (2) understanding and finding information about gout, (3) impact on identity, and (4) impact on roles and relationships.

**Conclusions:**

The diagnostic process for women with gout can be uncertain due to lack of awareness of gout in women (by health care professionals and women themselves). Women do not have a good understanding of the condition and find it difficult to find information that feels relevant to them. Gout has a major impact on women’s identity and on their roles and relationships. These findings are of importance to health care professionals dealing with women with potential gout and those with an existing diagnosis.

## Background

Gout is a common and extremely painful form of inflammatory arthritis, with a prevalence of around 2.49 % in the United Kingdom general population and 1.05 % in women [1]. In older women it is the most common inflammatory arthritis [2, 3]. Given the higher prevalence in men, most gout research has tended to focus on men. However there is evidence to suggest that older women are now increasingly experiencing gout [4]. It has been estimated that the incidence of gout in the United States has doubled among women over the past 20 years; in the UK prevalence in women increased by 4.6 % on average each year between 1997 and 2012; and in the UK incidence in women increased by 2 % on average over the same period [4].

Diagnosing and treating women with gout presents additional challenges to clinicians as women with gout are more likely to have atypical presentation (for example disease at the fingers and ankle) and present at an older age making treatment problematic due to co-existing comorbidities [5]. One study which explored people’s experiences of gout found that women in particular were unwilling to accept a gout diagnosis because of the associations with, and misperceptions of, the condition. They saw it is a ‘man’s disease’ and therefore chose to view it as an “alternative rheumatic disease.” [6] This finding is important, not least because “the myths and misconceptions” people have about gout can influence how it is viewed and treated [7]. Newspaper articles give an indication of the source of some of these myths and misperceptions: “Gout is agony, it's on the increase - and you don't have to binge like Henry VIII to get it” [8]; “It is usually regarded as a disease that strikes middle-aged men with a love of rich food and fine wine. But a new study highlights how a growing number of older woman are also suffering the excruciating pain of gout” [9]. The stories following these dramatic openings invariably include more references to Tudor monarchs, port and overweight men, even when the message of the story is that gout is not about these factors. Despite clinical evidence suggesting that gout is not (solely) a disease of rich, old men, it seems that media depiction draws on these aspects, and potentially contributes to current popular understanding.

We know that patient understanding of, and beliefs about, illness influence behaviour and that this behaviour may be informed by the media [10]. The mass media is a major source of information about health care and can also influence health care utilisation [11]. Women, and their healthcare practitioners, may be less likely to recognise their symptoms as gout, thus potentially delaying diagnosis. United Kingdom based research has shown that that if people consider gout to be self-inflicted they are less likely to visit the doctor and therefore put up with repeat attacks. They may also fear being criticised by their GP for lifestyle choices [6].

So, given the lower prevalence of gout in women compared to men, the often atypical presentation of symptoms, and the reluctance of women to accept a diagnosis of gout, all set within a context of continued media association of gout with rich living and male royalty, exploring the experience of women with gout is crucial. If women have a different experience of gout this may highlight opportunities where interventions could be better targeted to facilitate the accurate diagnosis and optimal management of this potentially curable disorder.

The aim of this study was therefore to gain a greater understanding of patient experiences, and to identify key features and issues within these experiences. We focus in this article on the experiences of the women in our study.

## Methods

### Design

A qualitative design was used. The study was granted ethical approval (NRES Committee South Central Berkshire 12/SC/0495 and 09/H0505/66) and written consent was obtained from all participants.

### Sample and recruitment

Maximum variation sampling (purposively selecting a heterogeneous sample) [12] was used to select a sample of 43 people, of whom 14 were women, from across the UK. A list of categories was created, using existing literature and expert advice (from a clinical and lay advisory group) to assist sampling. These categories included the types of experiences and demographic variables that were considered to be of most importance to clinicians and patients. Early recruitment was inclusive, using patients who responded to recruitment adverts to fill the most common categories (age, sex and years since gout diagnosis) with the only inclusion criteria being a self-reported diagnosis of gout being given from a healthcare practitioner, and a minimum age >18 years. As the study progressed, recruitment was targeted to ensure that all categories were covered. The aim of this method of sampling is that common patterns that emerge from a heterogeneous sample are of particular interest and utility in understanding the experience of living with gout [12].

We used a wide range of recruitment methods, including posters in general practices and rheumatology clinics, information packs handed out by members of the study advisory group, information on local and national gout support group websites, other online advertising and snowballing using personal contacts. Participants were contacted by telephone or email to arrange interviews.

### Data collection

An experienced qualitative researcher (JL) conducted face-to-face semi-structured interviews with participants. Interviews were audio or video recorded depending on participant preference. In order to encourage individuals to talk about their experience a narrative approach [13] was used. This meant that each individual’s own concerns, meanings and priorities could be explored, rather than these being structured by predetermined questions. The researcher used an interview guide to explore areas in more depth, and to ask about topics that had not been mentioned. One question in the topic guide asked specifically about the experience of gout as a woman (‘Do you think it makes a difference being a woman with gout?'). Interviews lasted between 25 min and 2 h, and took place in participants’ homes or workplaces.

### Data analysis

Interview recordings were transcribed verbatim and checked by the researcher (JL). NVivo 9 (QSR) computer software was used to facilitate data coding, sorting and retrieval. JL read and re-read all transcripts and constructed a coding frame of themes using the method of constant comparison, in which new data is compared with already collected data in order to refine the ‘labels’ given to themes in the data [14]. A second researcher (SP) checked these to identify any additional codes. Analytic themes were then discussed and developed further by the main authors (JL and JR) [15]. Further analyses and extracts from interviews are presented on http://healthtalk.org/peoples-experiences/bones-joints/gout/topics and illustrated with interview excerpts.

## Results and discussion

### Sociodemographic characteristics

The total sample included people from different age and ethnic groups, social backgrounds and geographical areas. This article uses data only from the female participants in the study (details of all participants can be found in another article arising from this study [16]). All names used are pseudonyms (Table 1).

**Table 1:** Sociodemographic characteristics of the women in the sample

	Women (%)
	(*n* = 14)
Age group at interview (years):	
30–49	3 (21)
50–69	7 (50)
70–89	4 (28)
Age group at diagnosis (years):	
<30	1 (7)
30–49	4 (29)
50–69	7 (50)
70–89	2 (14)
Time since diagnosis (years):	
1–5	6 (43)
6–10	4 (29)
11–15	3 (21)
≥16	1 (7)
Attacks in last 12 months:	
0	4 (29)
1–4	6 (43)
5–9	1 (7)
≥10	3 (21)
Ethnicity/Nationality:	
White British	13 (93)
Asian British	1 (7)
Geographical location:	
England	11 (79)
Scotland	2 (14)
Wales	1 (7)
Living arrangements:	
Living alone	4 (29)
Living with one other person	6 (43)
Living with more than one other person	4 (29)
Marital status:	
Married/Long-term partner	9 (64)
Single	2 (14)
Divorced/Separated	2 (14)
Widowed	1 (7)
Current work status:	
Retired	9 (64)
Full-time work	5 (36)

### Qualitative findings

Four themes emerged from the data in relation to experience and impact of gout as a woman: onset and diagnosis; understanding gout; identity; and roles and relationships. These themes obviously have overlap, but for clarity we present them separately.

#### Experience of onset, help-seeking and diagnosis

Misperception was a particularly strong theme in our study:

The GP looked at my foot […] and said, “If you weren’t a woman, I would say this is gout.” And I thought, “Ah, no, it can’t be,” because I had in my mind all of the old wives’ tales that we have in our minds about gout. And also I don’t drink excessively and all of the things that people associate with gout. And so I didn’t initially take that throwaway comment that seriously. (Joanne)

So I went to the GP and explained all the symptoms and she said, “Well I don’t think you’ve got gout but I’d better test you for it because your symptoms are sort of connected with that.” And she said, ‘Well it's very unlikely because-female, and your age, and you’re not particularly overweight, or anything, but we’ll test anyway.” (Georgina)

Joanne and Georgina are both women who actually went to the doctor with symptoms, but for others the stereotype of it being a man’s disease may prevent them from even getting that far.

Erm, I suspect, I suspect people might be surprised. As I said earlier, I think it may very well be that some women go undiagnosed because it’s not spoken of as something-as a condition that women can be diagnosed with. (Joanne)

For Lily, a younger woman, the process of diagnosis felt uncertain:

No doctor, no medical professional. Because I think-I’m not sure how much GPs know about it, to be honest. And I’m not trying to belittle them. It’s just that it’s not one of those red flag things that, “You have to read this.” And then I’m also not erm a very representative example of someone who has gout that they have to worry about. I’m not a 70-year-old Indian man or an 80-year-old English man, for that matter. I’m, I’m young and, and a female doesn’t really come that often to them so I don’t really think they know much about it. They don't, I think. (Lily)

Lily’s description of her experience as a young woman with gout reflects that fact that GPs do not see many patients in this category.

For the women in our study, the shock of an unexpected diagnosis of gout led them to want to know more information about the condition.

#### Understanding of and finding information about gout

Women in our study reported knowing little about gout before being diagnosed:

I’d be interested to know if there were people a lot younger than I was. Perhaps if it is connected to the menopause or to age, or age related for women, or perhaps because of the oestrogen or something like that.

Had you heard of that [gout more common after menopause] before I just mentioned it? I had vaguely, but I’d forgotten, yeah. But it’s never something that’s particularly prominent when you read articles or anything (Judith)

For younger women, stereotypes of age and gender provide a particular context in which they attempt to understand their own experience of gout:

I just thought it was old men that drank Port that got it in their big toe. Err I knew that it was painful, but I never thought a young woman who drank pints of lager could get it. Err no I don’t know. I don’t know. It’s one of these once you hear about … until you’re diagnosed with it you don’t really know. (Mary)

Finding information that feels relevant as a woman was reported as an issue:

Well, it just happens, doesn’t it? There’s nothing you can do about it. It, it, the stereotype is there, and it may be quite wrong. I mean, you, you never see pictures of, erm, elderly ladies with their feet up on stools and swathed in bandages. It’s always these portly gentlemen, isn’t it? It, it’s, it’s just the, sort of, tradition that’s been handed down, and it’s a pity because gout has become the butt of people’s humour, when really it is quite a serious and painful ailment. (Cheryl)

I think…it’s not normal kind of thing. Because you do look at everything and you know the pictures, any of the joint pictures, you can tell they’re men-either that or really hairy women [laughs] but-and you think well you know that can’t be erm…have they made a mistake kind of thing. I know it’s not a mistake but I am the only woman, as far as I know, out there with it […] I am just intrigued to know why me really. Because I don’t fit the mould as-as such. I am a freak of nature. (Georgina)

The lack of relevant information clearly has an impact on how Georgina feels about herself and about her condition.

When the stereotypes of age and gender interact, things become challenging, particularly for young women. For men, the issue is often that their age and gender are aligned with the stereotypical characteristics, whereas for women, the opposite applies. Women may have other issues as a result of gout that are unique in terms of their gender. For example, one of our participants who wanted to conceive found it difficult to find accurate information regarding gout and pregnancy, even including medication safety. For her, gout was delaying what she saw as the next stage in her life. In contrast, for Georgina it is simply about not recognising herself in the information that is available and the impact that has on her identity as a woman:

I think so, yeah. Because you-you don’t associate-you associate osteoarthritis, osteoporosis, more with women, but you wouldn’t really-well in anything I’ve seen, you wouldn’t associate women as having gout. So I think it is-I mean it’s obviously quite rare because a lot of the data you look at is the same sort of thing, you know, that you’re more likely to have it if you’re overweight and male and fifties and…so I would say it’s very erm…very different, and quite-a bit more discriminating towards women, because you don’t fit the bill. So I erm and then I suppose it’s not-I would assume, there’s not that many women out there because you can’t find a lot of information on women with gout. And what impact it can have. Apart from the general information then. (Georgina)

Some of the participants found online forums useful, for their role in sharing experiences with other women:

But from a woman’s point of view, get on the forum and try and get in touch with people, especially women who’ve got, because they are going to be more understanding. Because especially from my point of view the men that I’ve come across with it, have abused their bodies, and know then themselves they’ve got gout through doing what they’ve done, so I think-but from - normally from a woman’s point of view, you know you - you don’t drink as heavily as men and that and because it’s more that we’ve always thought it was connected to drink, but I just think it’s beneficial for women to be in touch with women who’ve erm-who’ve developed it-definitely. (Georgina)

The focus on men and, what the women perceived as, self-inflicted factors, and the overall scarcity of relevant information for these women diagnosed with gout created severe challenges to their identity, epitomised by Georgina’s comment that she felt like a “freak of nature”.

#### Impact of gout on identity

The following excerpts provide further powerful examples of the effect of the symptoms of gout on a woman’s identity, namely the presentation of self as feminine through footwear:

I: And, do you think it makes a difference being a woman with gout?

R: Probably because of the shoes. Yeah, probably does. Because you do feel more mannish I think. Although I shouldn’t worry at my age should I? If you’re 19 up here [taps head] and you’re not this old bat, you know?

I: I don’t think, you know, I think you’re a woman at any age aren’t you?

R: Yeah but, you know, society like, you do get to that invisible age where people just don’t see a middle aged woman anymore. But in your head you’re still that young girl, you know, with that young girl’s feelings.

I: And is that something that you think having gout and having to sort of change your footwear, do you think that sort of err moved you towards that?

R: Yes, yeah, definitely. I did, I mean, and I noticed all the other women’s footwear at the wedding and there was loads of really, really high shoes. I mean mainly young girls but there were some really classy shoes there and I thought, ‘That’s me goodbye forever’. You know. I knew that really and, like I say, when I watch the television and I see the girls on the television come out in these sort of four, five heel, inch heels, and I think, ‘Oh I wish’. [….] … it’s part of who you are isn’t it? (Wendy)

If I do have to go out of the house, I’ve got a huge pair of flip flops on, I’m shuffling, and it’s very uncomfortable. To be honest with you, [researcher’s name], I wouldn’t go out of the house, it would be too uncomfortable. You […] cannot wear your shoes. And then people start to look because you’re shuffling, it’s uncomfortable. I’m usually pulling my face because I’m in pain. [laughs] I would tend not to go out of the house at all, that’s why I say it’s a disabling disease to me. (Mandy)

Wendy’s poignant description of being unable to wear the shoes that she likes is resonant of research on other chronic disabling conditions, particularly rheumatoid arthritis, where research suggests that the constraints made by lack of suitable footwear choices can contribute to feelings of dissatisfaction with body image and low self-esteem [17].

The actual term ‘gout’ was problematic for some women in our study, because of its impact on their identity. Some participants reported telling people they had arthritis rather than gout, as found in a previous study, where participants presented gout as “an alternative rheumatic disease” [6] The inability to wear ‘normal’ footwear makes a largely invisible condition visible and opens people up to the potential stigma associated with a ‘disabled’ identity, as expressed by Mandy.

#### Impact on roles and relationships

Identity was also affected by the impact of gout on roles and relationships. The impact of gout on this area for women was similar to that for men. Women reported that their work was affected, although they were also able to make adjustments, for example, provision of special equipment, or being able to work from home during an attack. One participant works as a volunteer because it gives her greater flexibility to take time off during attacks. She did not believe that this would be possible if she was in paid work, while another felt that having regular flares of gout meant she could not look for a new job because it would be too difficult to explain her situation to a new employer.

And I suppose in your foot, because where you’ve got to have your feet to get out and about, I mean it’s - sometimes if it’s in your hand you can rest that but you can’t rest your feet. And in work if I’ve had a particularly busy week I come home on a Friday and the elbow’s painful, the hand is painful, but I wouldn’t class that as an episode as such because I always class that initial one, I sort of scale them all compared to that initial one and they’re nothing like I had then, so I’m thinking that’s just part of it, you know, you’ve just got to deal with it. Take a couple of painkillers and then normally the following day… so on the weekend I think my husband’s doing more of the manual cleaning than I used to, because I do find that a bit of a struggle, and he’s marvellous and he’s taken on a lot of the ironing erm chores and things because I find that difficult sometimes, it’s the constant with the iron and that, but erm I just try and get on with things really. (Georgina)

The description by Georgina of the impact on her work outside and inside the home illustrates the effects on both areas, and the impact on other members of her family, which she elaborates on further:

So erm I think it does impact everybody really. It’s been an impact on my family because I - I don’t over exert myself and I’ve always been the type of person that I - I won’t ask anyone to do something because I’d rather do it myself. You know, the self-satisfaction there of doing it yourself. And to have, you know, my husband take on a lot of responsibilities, for instance now bending over and cleaning the bath, like if you’ve got to hold on with one hand, and clean with the other, you know, I don’t like risking that either. And he often jokes, ‘Oh, you’re only putting it on now because …’ But I don’t like asking people to do things for me, so I do get frustrated quite a bit. And erm with the kids, when you’re in pain sometimes you know you - you’re quite sharp with the kids and they’re sort of looking and you’ve got to explain then you know but…especially for my 13 year old, she doesn’t know - really know what’s going on half the time. She doesn’t even know what the condition is I suppose […]. And it’s the fear of the unknown because always in the back of your mind that you’re going to - is it going to be worse? Am I going to have a flare up?, and so you’re over cautious then. (Georgina)

A predominant theme throughout the accounts was the impact of gout on relationships with families or friends. Mood was affected by their symptoms and pain, and this could be difficult for other family members to deal with. Others talked about wanting to be left alone during attacks. Being understood by friends was felt to be important:

Well luckily the people that come to look after me as it were, they know that I can get - it does, it makes you irritable because it’s painful and it’s - it’s uncomfortable and you want to go to the loo but you know that simply just by getting up and shuffling to the loo, it’s like going to be a main thing to do. And it’s going to be painful to do. So people do come and I am ratchety, but they know it’s because I’m in pain. (Mandy)

Again, as with men’s experience, the physical and sexual aspects of people’s relationships were also affected. People did not want to be touched because they were worried about pain. However one woman described the pain relief she got from orgasm:

Erm because then err we’ve noticed that in a month’s cycle I’m more likely to get gout or an attack the week before I have my period, which is also the week wherein it’s like the end of fertility, the highest fertility, which is when erm I guess there is more desire for sex. [hmm] And bodily as well, the fluids are there, etc., etc. And then that is when you have the gout, so it just - it doesn’t really work very well like that, because then you cannot do much, but, but that’s when it happens and then so it doesn’t really work very well. No, but it does help pain relief, which is weird. (Louise)

## Conclusion

This study provides evidence about women’s experience of gout, in the context of diagnosis, understanding and learning about gout, and impact on identity, roles and relationships.

The key messages from this research are that the diagnostic process for women can be uncertain due to lack of awareness of gout in women (by health care professionals and women themselves). All of the women in our study self-reported a diagnosis of gout by a health care professional. (Although we were not able to verify this diagnosis, previous studies have found that this is reliable [18]). Women do not have a good understanding of the condition and find it difficult to find information that feels relevant to them, as much is targeted at men. Gout has a major impact on women’s identity and on their roles and relationships. These findings are of importance to health care professionals dealing with women with potential gout and those with an existing diagnosis.

The participants in our study did not express that they experienced stigma on the basis of their gout per se. Any stigma that exists in the context of gout seems to be about the outward signs/ lack of outward signs (hence behaviour) that gout occasions rather than about the gout itself. In that sense people’s experience is similar to that of other chronic illnesses which are largely invisible and episodic. The reactions of other people in response to participants’ gout could be said to be stigmatising, based as they are on negative stereotypes - but not all participants experienced it as such, rather as an experienced stigma associated with being unable to fulfil expected roles or a loss of self and identity for the same reason, as with Wendy. People may use strategies of controlled information release [19] in order to mitigate social stigma and maintain positive identities as far as possible within the cultural milieu in which they operate [20]. Controlling the release of information means not only deciding who to tell but also what kind of information to give. For some women with gout, this means using a general term such as ‘arthritis’.

Two recent analyses of gout education resources suggest that problems exist with such resources in terms of lack of readability, important missing information and poor usage of key messages [21, 22]. Neither of these studies explored the issue of gender in patient resources but we would argue, from an analysis of our data, that key messages would include those that are relevant to women with gout. It is also important that future research relating to gout includes women’s experiences.

One of the limitations of our study is that the participants were all volunteers, recruited through a range of strategies. It is possible that other people with gout may have different views, for example, future studies could target women in the ‘oldest old’ age group.

The importance of understanding more about women’s experience of gout is that women may not currently be getting optimal treatment in that diagnosis may be missed and treatment not started promptly. The unexpected nature of a gout diagnosis for women, particularly if this diagnosis also appears unexpected to the doctor, can lead to a lack of confidence in this diagnosis. Dealing with the unexpected nature of the diagnosis, especially in terms of gender is crucial to treating the condition. If gout and its implications are recognised, by women and health care professionals, then any problems can be dealt with effectively. A study in New Zealand found that women who were treated were more likely to be at target level for treatment of gout, which the authors suggest may be due to comparatively lower serum urate levels in women, or to different health care utilisation behaviour [23]. This underlines the importance of diagnosing and beginning treatment of gout in women.

### Ethical approval

NRES Committee South Central Berkshire 12/SC/0495 and 09/H0505/66.
